# Experimental Study of Potential CD8^+^ Trivalent Synthetic Peptides for Liver Cancer Vaccine Development Using Sprague Dawley Rat Models

**DOI:** 10.1155/2022/4792374

**Published:** 2022-05-31

**Authors:** Sidra Zafar, Baogang Bai, Jinlei Guo, Syed Aun Muhammad, Syeda Tahira Qousain Naqvi, Muhammad Nauman Shabbir, Imran Imran, Rehan Sadiq Shaikh, Amjad Ali

**Affiliations:** ^1^Institute of Molecular Biology and Biotechnology, Bahauddin Zakariya University, Multan, Pakistan; ^2^School of Information and Technology, Wenzhou Business College, Wenzhou, Zhejiang, China; ^3^Engineering Research Center of Intelligent Medicine, Wenzhou, Zhejiang, China; ^4^The 1st School of Medical, School of Information and Engineering, The 1st Affiliated Hospital of Wenzhou Medical University, Zhejiang, China; ^5^School of Medical Engineering, Sanquan College of Xinxiang Medical University, Henan, China; ^6^Department of Statistics, Bahauddin Zakariya University, Multan, Pakistan; ^7^Department of Pharmacology, Faculty of Pharmacy, Bahauddin Zakariya University, Multan, Pakistan; ^8^Centre for Applied Molecular Biology, University of the Punjab, Lahore, Pakistan; ^9^Atta-ur-Rahman School of Applied Biosciences (ASAB), National University of Sciences and Technology (NUST), Islamabad, Pakistan

## Abstract

**Background:**

Liver cancer (LC) is the most devastating disease affecting a large set of populations in the world. The mortality due to LC is escalating, indicating the lack of effective therapeutic options. Immunotherapeutic agents may play an important role against cancer cells. As immune cells, especially T lymphocytes, which are part of cancer immunology, the design of vaccine candidates for cytotoxic T lymphocytes may be an effective strategy for curing liver cancer.

**Results:**

In our study, based on an immunoinformatics approach, we predicted potential T cell epitopes of MHC class I molecules using integrated steps of data retrieval, screening of antigenic proteins, functional analysis, peptide synthesis, and experimental *in vivo* investigations. We predicted the binding affinity of epitopes LLECADDRADLAKY, VSEHRIQDKDGLFY, and EYILSLEELVNGMY of LC membrane-bounded extracellular proteins including butyrophilin-like protein-2 (BTNL2), glypican-3 (GPC3), and serum albumin (ALB), respectively, with MHC class I molecules (allele: HLA-A∗01:01). These T cell epitopes rely on the level of their binding energy and antigenic properties. We designed and constructed a trivalent immunogenic model by conjugating these epitopes with linkers to activate cytotoxic T cells. For validation, the nonspecific hematological assays showed a significant rise in the count of white blood cells (5 × 10^9^/l), lymphocytes (13 × 10^9^/l), and granulocytes (5 × 10^9^/l) compared to the control after administration of trivalent peptides. Specific immunoassays including granzyme B and IgG ELISA exhibited the significant concentration of these effector molecules in blood serum, indicating the activity of cytotoxic T cells. Granzyme concentration increased to 1050 pg/ml at the second booster dose compared to the control (95 pg/ml), while the concentration of IgG raised to 6 g/l compared to the control (2 g/l).

**Conclusion:**

We concluded that a potential therapeutic trivalent vaccine can activate and modulate the immune system to cure liver cancer on the basis of significant outcomes of specific and nonspecific assays.

## 1. Introduction

Liver cancer is the sixth most prevalent type of cancer around the world [1]. Countries where people ages 20 years or less are suffering from this disease are considered high risk, while countries are at lower risk where LC is less prevalent in people of age 50. The available treatment for LC includes chemotherapy, radiotherapy, liver resection, and laparoscopic hepatectomy [2]. These treatments may show negative effects on normal cells and may cause reoccurrence of cancer, toxicity issues, and expensive procedures [3]. Medical specialists and oncologists have some concerns over conventional procedures [4]. Although these treatments are available options, still there is a need for safe and alternative therapeutic strategies.

Immunotherapy can be the best frontier for the treatment of cancer, and it is considered valuable and effective in curing liver and other cancers. Although a number of recent developments have been made in this area, however, still we need further investigations to design therapeutic vaccines for liver cancer [5, 6]. Immunotherapeutic peptides boost up the body's natural immune system to fight against cancer cells. The efficacy of immunotherapy can be recognized by the fact that for the destruction of tumor cells, they use immune components. Therefore, immunotherapy is considered an important alternative for LC treatment [7]. To understand the pattern of liver cancer proliferation, some important regulatory immune components like CD8^+^ and CD4^+^ T cells, NK cells, myeloid-derived suppressor cells (MDSCs), and regulatory T cells are some types of cells that are studied in detail. On activation, these lymphocytes provoke other immune system components and activate either cell-mediated or humoral immunity or both [8]. Immunological facts of an immune response are now explored and understood by a basic scientific approach with computational tools [9]. Whole-genome analysis contributes to the development of vaccine candidates that will fight against specific targets. Currently, a newly emerging technique, reverse vaccinology, instead of using the culture, only requires the genomic sequence of the target protein, which is further utilized for vaccine preparation [10, 11]. The complete genomic sequence helps in the prediction of potential target epitopes, and vaccines can be designed against the respective organism [10, 12].

New computational tools, software, and genomic and proteomic databases are an important part of immunoinformatics and reverse vaccinology approaches for the prediction of specific epitopes. Hence, immunoinformatics is a computational study related to biological compounds, their modeling, and complications of the immune response. These integrated techniques provide the basis of vaccine development [13] involving analysis of genomic to proteomic sequences, identification of B and T cell antigenic sites, and cellular responses [14] until there is no approved prophylactic and therapeutic vaccine for liver cancer [5, 15]. However, it has been reported that immunotherapeutic vaccines for liver cancer are under preclinical or clinical trials but not yet approved [16]. In this investigation, our main objective is to seek out the LC-specific epitopes to provoke an adaptive immune response. With the help of software, online servers, and databases, we designed specific T cell immunogenic peptides. The efficacy of these immunogenic peptides was evaluated by hematological assay, antibody-specific (IgG) assay, and granzyme ELISA. For this purpose, *in vivo* experimental trials were carried out on the Sprague Dawley rat model. This study describes the significance of immunoinformatics for the prediction of liver cancer epitopes and the efficiency of predicted potential immunogenic candidates by *in vivo* trials.

## 2. Methodology

### 2.1. Ethical Approval and Study Design

**Figure 1:**
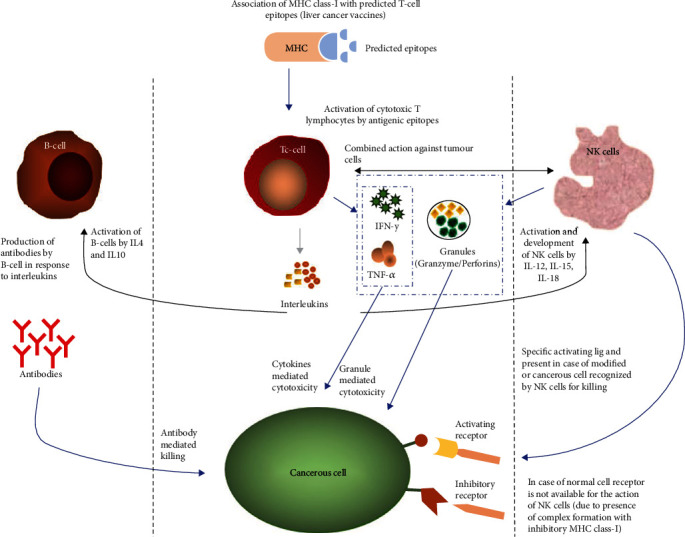
Schematic representation of our hypothesis.

**Figure 2:**
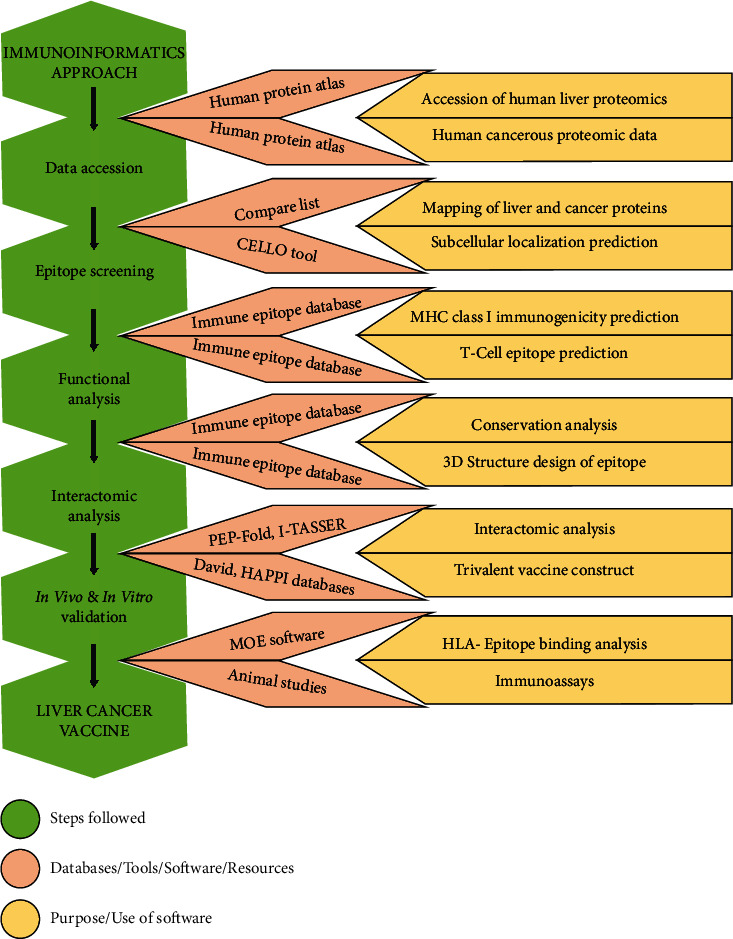
The integrated steps of our framework used for the prediction of T cell epitopes.

All animals were kept under standard conditions as per ARRIVE guidelines reported for animal handling. These experimental procedures were approved by the Bioethics Committee for Animals of the Institute of Molecular Biology and Biotechnology, Bahauddin Zakariya University, Multan, Pakistan, with Approval No. IMBB/02/2019. In this study, we predicted the T cell epitopes of cytotoxic T lymphocytes (CTLs) (CD8^+^ cells) based on an immunoinformatics approach [17]. These epitopes were docked to MHC class I molecules to analyze the binding affinity. Based on the binding energy, three epitopes were linked to construct a potential trivalent vaccine candidate. Our hypothesis involves the activation of CD8^+^ cytotoxic T lymphocytes (CTLs) as they have MHC class I receptors to boost up the natural immune system to target cancerous cells. Granzymes and other chemical mediators are released upon activation of CTLs [18]. These granzymes and chemical molecules have a significant role in the apoptosis of cancer cells. Granzyme B is secreted from CTLs and NK cells; according to our hypothesis, secretion of granzyme B in immune cells may help in killing the cancerous cells. IL-4 and IL-10 released by a subpopulation of CTLs activate B lymphocytes resulting in antibody production [19–21]. The hypothesis of our study reflects this mechanism, and animal investigation indicated that our trivalent molecule is antigenic in nature, which is potentially able to activate the B and T lymphocytes (Figure 1). In this study, we focused on tumor-specific and tumor-associated antigens to report newly designed potential epitopes as immunotherapeutic agents for liver cancer. For screening of liver cancer antigens, we used the TRON Cell Line Portal [22] and Tumor-Specific Neoantigen Database (TSNAdb) [23] to give a specific category to each protein. Moreover, these proteins also have high expression in the liver. With the help of the GenomicScape server, these proteins were further tested for their expression levels in the liver [24]. The system-level analysis of these peptides showed the significant level of production of antibodies, interferons, granzymes, and other mediators. The study framework was designed based on integrated steps (Figure 2) using different bioinformatics tools, databases, and software, *in vivo* (Table 1).

### 2.2. Screening of Liver Cancer-Associated Proteomic Data

The Human Protein Atlas database was used for the retrieval of liver cancer-associated proteins [25, 26] (Supplementary Table 1 and 2). We used the compared two-list procedure of the Whitehead BaRC public tool to screen liver cancer proteins from all cancer data. The FASTA sequences of screened proteins were accessed from the UniProt Knowledgebase (UniProtKB) database [27]. The extracellular and membrane-bound proteins were predicted by the CELLO subcellular localization predictor tool [28].

### 2.3. MHC Class I T Cell Epitopes and Immunogenicity Prediction

The T cell epitopes of liver cancer-associated antigenic proteins were predicted using the Immune Epitope Database (IEDB) and selected based on the lower percentile rank (0.1%) cutoff parameters [29]. The predicted T cell epitopes showed the connotation of the HLA-A∗01:01 allele of MHC class I molecules expressed in the maximum number of populations. The conservation of these epitopes was determined by IEDB [30] indicating the optimal allelic binding affinity [17, 31].

The ability of an epitope used as a vaccine candidate depends on the features of antigenicity and immunogenicity, and these were studied by the VaxiJen server and IEDB [32]. The ExPASy Compute pI/Mw tool was applied for molecular weight prediction [33].

### 2.4. Physicochemical Properties

The physicochemical properties of the peptides were predicted using online servers [34]. Tools PEPlife, ToxinPred, ExPASy, Peptide 2.0, and PredSTP were used to predict physicochemical properties [35–37]. Half-life is the time taken by half the amount of protein to remove from the body after entering the cell. Stability of a peptide in the test tube or in the synthesized drug form is indicated by the value of the instability index. For a protein to be soluble in water, it must have a charge on it. Detailed knowledge of the hydropathicity of an individual amino acid enables us to predict the overall three-dimensional structure of a protein from its amino acid sequence. Toxicity profiles of peptides must be predicted unless we cannot use them in clinical trials. The SVM (Support Vector Machine) model of amino acid composition was used to predict the toxicity.

### 2.5. Construction and Modeling of Trivalent Molecules

The HADDOCK server was used to predict the ranking and best binding combinations of epitopes based on an algorithmic score [38]. The refinement interface of this server was used to get the refined C port modules of epitopes and their active and passive residues [39]. Suitable linkers were applied between epitopes to avoid unwanted joining and denaturation of the trivalent constructs. The functionality of the trivalent construct and the arrangement of epitopes also depend on these linkers [40]. AAY is the most commonly used linker for HLA-I epitopes [41, 42]. The trivalent construct was modeled using the I-TASSER server [43], and the quality of configuration of the trivalent model was defined by a 3Drefine tool [44]. Molecular Operating Environment (MOE) software was used for the estimation of the binding energy of a trivalent multiallelic molecule to MHC class I receptors based on the Root-Mean-Square Deviation (RMSD) and binding energy (*E* score). For verification, the potential epitopes were analyzed and assessed using various software and tools.

### 2.6. HLA Epitope Binding Analysis

The 3D model of HLA-I (MHC class I) was accessed from the Protein Data Bank (PDB). The binding affinity between predicted T cell epitopes (trivalent model) and HLA molecules was carried out by molecular docking analysis using MOE software.

### 2.7. Synthesis of Trivalent Peptides

The trivalent construct (LCPV09) with >95% purity was synthesized commercially by Shanghai Research Institute of Chemical Industry (Shanghai TECH. Chemical Industry Testing Co., Ltd., China).

### 2.8. Animal Immunization

Animals were obtained from the breeding unit of the Department of Pharmacology, Faculty of Pharmacy, Bahauddin Zakariya University, Multan, Pakistan. Preclinical trials are performed on animal models for the validation and optimization of peptide doses [42] and experimental trials. After screening and optimization of vaccine candidates, human tissues/hepatic cell lines and disease animal models are used in the next step. Efficacy analyses are performed on animal models for the validation and optimization of peptide doses. Sprague Dawley rats of weight 150 grams, 5-6 weeks old, were used. Rats were divided into four groups (*n* = 6/group). One group of rats was immunized with a 150 *μ*l solution of 160 *μ*g of trivalent molecules alone. The second group was given 150 *μ*l of an adjuvant which is aluminum hydroxide solution (G-Alum™ Adjuvant Kit, Cat. # 786-1216), and the third group was immunized with a combined solution of trivalent molecules plus 2 times of aluminum hydroxide solution. The fourth group was taken as the control group, and nothing was given to those rats. The doses were injected into the lower abdomen subcutaneously. A first booster dose of 240 *μ*g and a second booster dose of 320 *μ*g were given with the same pattern to each of the four groups to get the optimized results. After immunization of rats with different doses, they were kept under observation for 2-3 weeks. Three weeks postimmunization, blood samples were taken from rats for immunoassay. Blood samples were collected by rupturing the vein in the eyes of rats using capillaries of 75 mm length and 1.1-1.2 mm inner diameter.

### 2.9. Hematological Assays

Serum was separated from blood by centrifugation in a gel clot vial. 150 *μ*l sample serum was added to the detection buffer and mixed by inverting the tube 10 times. Two drops (75 *μ*l) of the sample mixture were dispensed in the sample cartridge. The cartridge was loaded into the reader immediately. After scanning, the CRP reading was displayed on the screen of the reader. The results were expressed in mg/l. Similarly, the levels of total white blood cells (WBC), lymphocytes (LYM), and granulocytes (GRA) in the blood of immunized rats were analyzed by Beckman Coulter, USA. The Coulter principle was used for the counting of WBC, LYM, and GRA in the blood. The results were observed in either percentage (%) or 10^9^ cells/liter [45].

### 2.10. Liver Function Tests

Liver function tests (LFTs) including serum alkaline phosphatase (ALP), alanine transaminase (ALT), aspartate transaminase (AST), and alpha-fetoprotein (AFP) were performed to detect the integrity of the liver of immunized rats and to observe the liver toxicity.

### 2.11. IgG ELISA

Serum samples of each group were analyzed for IgG after three weeks of immunization using the ELISA kit as per the manufacturer's guidelines (Roche: Mouse IgG ELISA) and the microplate reader (BioTek 800TS). Specific ELISA was performed using the Antigen-Down ELISA Development kit in which the LCPV09 antigen was coated in the wells to quantify the IgG. Its ELISA-based technique is in which a specific antigen of animals is coated on wells to record the IgG. The coating reagents (Na_2_CO_3_ buffer 0.95 *w*/*v* of 0.5 M sodium azide at a pH of 9) with 50 *μ*l of the capturing antibody in the final concentration of 25 *μ*l/ml were dispensed in wells with appropriate dilution. The microwell plate was incubated for 90 minutes, and after incubation, the solutions were discarded and dried on cellulose paper. 200 *μ*l of the washing solution which contains 0.9% *w*/*v* NaCl and 0.1% *v*/*v* Tween-20 was used to wash the wells. 200 *μ*l of the 1x blocking reagent (1 : 10) containing gelatin powder, NaCl, and Tris-HCl buffer was added and incubated for 90 minutes. Then, the solutions were removed and a 200 *μ*l solution of antibodies extracted from serum at a concentration of 25 ng/ml was added to a microtiter plate. Steps of removing the solution and washing were repeated after incubation. The anti-mouse (POD) conjugate solution of 50 *μ*l along with 1 ml of the blocking reagent was poured into the wells. The wells were incubated and washed. Finally, 50 *μ*l of the substrate (2,2′-azino-bis(3-ethylbenzothiazoline-6-sulfonic acid)) was added and the fluorescence was recorded at 492 nm to analyze the enzyme-substrate interaction indicating the concentration of IgG in samples (g/l). The mean value ± SD of IgG of control rats was taken as the standard [46].

### 2.12. Granzyme B Assay

The blood of immunized rats was analyzed for granzyme B concentration. Serum was separated from whole blood by using a gel clot vial. ELISA was performed by using the Granzyme B Platinum ELISA kit (Thermo Fisher Scientific, Catalog # BMS2027) according to the manufacturer's guidelines. The first step was washing the microwell plate twice by using the 400 *μ*l washing buffer (PBS with 1% Tween-20). Wells were dried after 10-15 seconds of washing by tapping a paper towel. The microwell plate was used immediately after washing and drying. 75 *μ*l of the dilution buffer was added in duplicate to sample wells (A1/A2) and 100 *μ*l to standard wells (B1/2, G1/2) in duplicate. Wells A1/2 were then filled with 75 *μ*l of the prepared standard solution of granzyme B. Solutions of the wells were mixed carefully so that the base of the wells must not get scratched. 50 *μ*l solution was transferred into wells B1/2. This process was continued until two rows of wells were covered with a standard solution of granzyme B ranging from 480 to 0.7 pg/ml. 50 *μ*l of the solution from each well was disposed of. 100 *μ*l of the dilution buffer was added to blank wells and 50 *μ*l to sample wells in duplicate. Each sample solution of 50 *μ*l was dispensed into sample wells. Adhesive film was used to cover the wells and incubated for 60 minutes at 15-25°C. The plate was placed on a shaker at 400 rpm. Adhesive film was removed after incubation time, and all wells were washed with 100 *μ*l of the biotin conjugate. After incubation, 100 *μ*l of the streptavidin-HRP dilution was added to all wells. Adhesive film was used to cover the plate, and an incubation time of 30 minutes was given at 18-25°C with shaking at 400 rpm. After that, the wells were washed carefully and 100 *μ*l of TMB as the substrate was added to all wells. After incubation of 15 minutes, color was observed in each well. Upon dark blue appearance, 100 *μ*l of the stop solution was added to stop the enzyme reaction. Stop solutions must be distributed equally throughout the well. The microplate reader (BioTek 800TS) was used to examine the absorbance of each well at a primary wavelength of 450 nm and a reference wavelength of 620 nm. The concentration of granzyme B was calculated and recorded in pg/ml. The standard was determined by the mean estimation of granzyme B of control rats' serum ± SD [47].

### 2.13. Statistical Analysis

Statistical analysis was performed on the mean values of the results of all replicates with standard error of the mean (SEM) and standard deviation (SD). Mean values of immunized groups were compared with the control group to check the level of significance using Minitab software. To carry out data interpretation, one-way analysis of variance (ANOVA) was calculated by using SPSS software. *p* value less than 1.0 × 10^2^ (>0.01) is statistically significant ± SEM [17, 48].

## 3. Results

### 3.1. Screening of Liver Cancer Proteins

**Figure 3:**
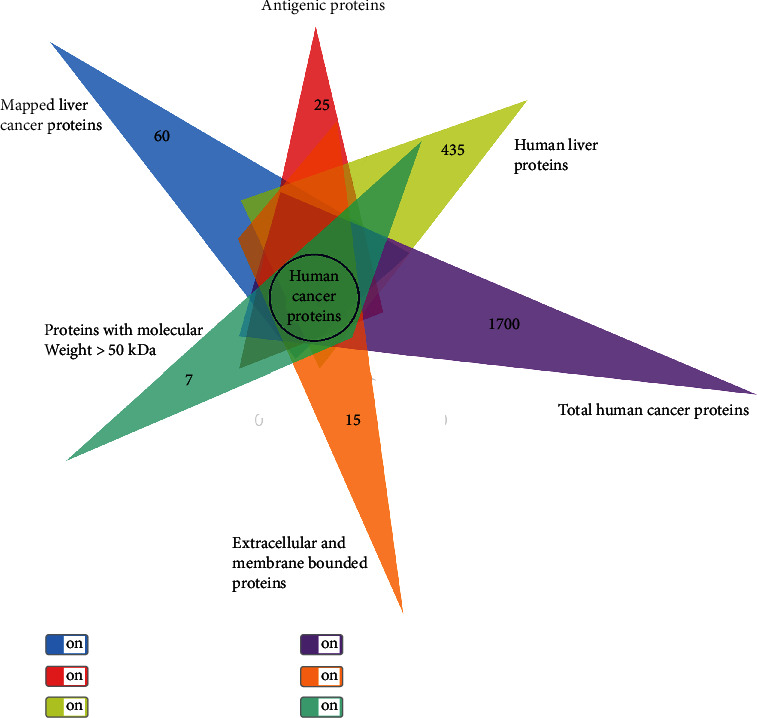
Screening of liver cancer-associated proteins for the prediction of antigenic peptides.

We screened 60 LC-associated proteins from a total of 1700 cancer proteins and 435 liver proteins. The proteins with high molecular weight were antigenic, having the antigenicity threshold score of 0.5 [32]. Based on the antigenicity score and molecular weight (>50 kDa), three proteins including ALB, GPC3, and BTNL2 were selected (Figure 3). Subcellular localization showed that ALB protein is present in the extracellular matrix, while GPC3 and BTNL2 proteins are membrane bounded. These proteins are tumor-specific proteins, and they are expressed only in the case of liver tumors (http://biopharm.zju.edu.cn/tsnadb/). All genes and proteins of the body are interlinked with each other in other organs of the body directly or indirectly. We screened the proteins on the basis of their optimal expression in the liver. These three proteins have the maximum expression levels in the liver or fetal liver (http://genomicscape.com/microarray/expression.php). ALB is a liver tissue enriched gene expressed in the right lobe of the liver (UniProtKB). There are many reported liver diseases associated with this protein. Serum albumin is mainly plasma protein and produced by the liver. Levels of this protein were checked in blood analysis for diagnosis of liver diseases. There are many therapeutic uses of albumin in liver diseases [49]. Clinical trials have evaluated that glypican-3 (GPC3), a tumor-specific antigen, is expressed in all hepatocellular cancers. It is usually found anchored to a protein expressed on the cell surface known as glycosylphosphatidylinositol (GPI). Glypican-3 (GPC3) is a promising target for anticancer immunotherapy against HCC even in its early stages because its expression is specifically observed in >80% of HCCs [50]. A peptide vaccine prepared from GPC3 was efficacious and safe in promoting tumor infiltration by cytotoxic T cells [5]. BTNL2, a butyrophilin-like protein, has been found to regulate the immune response. The expression of BTNL2 has been linked to sarcoidosis in the liver [51, 52]. It has been shown in several *in vitro* studies that B cells have surface receptors for BTNL2, while CD8^+^ and CD4^+^ cells express such receptors upon activation. Thus, it can be employed as a therapeutic agent to reestablish immune tolerance in such inflammatory conditions. Furthermore, BTNL2 can be modulated to arbitrate the immune response for developing innovative therapeutics if the relationship between its structure and function can be fully illustrated at the molecular level [51].

### 3.2. T Cell Epitope Prediction

T cell epitopes complexed with MHC class I are strongly immunogenic and induce the immune system robustly. The predicted T cell epitopes of BTNL2, GPC3, and ALB were EYILSLEELVNGMY, LLECADDRADLAKY, and VSEHRIQDKDGLFY, respectively. These potential T cell epitopes have the potential to target the maximum number of alleles indicating conservation in the entire population. Such multiallelic epitope-based vaccine candidates have more spectral features compared to those candidates that target one type of allele.

### 3.3. Physicochemical Properties

Half-lives of epitopes are linked to their stability as well as toxicity. The half-life of a good epitope must be less than hours to make it stable and to activate the immune components. Half-lives of our predicted epitopes were less than 10 hours presenting stability and less toxicity. The instability index also measures the stability of the peptide [52]. These epitopes have a less instability index presenting the stable drug form. Our epitopes showed water solubility, and amino acids are either hydrophobic or hydrophilic. The lesser value of hydropathicity means a more hydrophilic character of epitopes. Almost all predicted epitopes have a less value of hydropathicity [53]. The algorithmic SVM score of the software was used to classify the epitope as being either toxic or nontoxic (nontoxicity threshold value < 5) [37, 54]. The predicted epitopes are nontoxic, having an SVM score of less than 5 (Table 2).

### 3.4. Trivalent Construct against Liver Cancer

**Figure 4:**
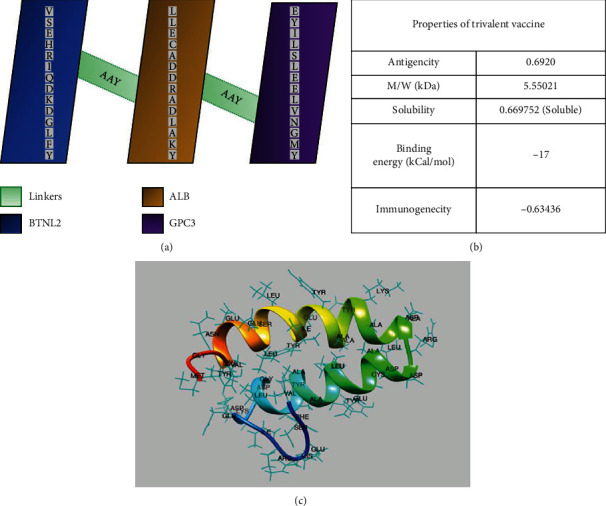
Schematic representation of the trivalent construct. (a) Trivalent contains 48 amino acid residues. MHC class I-predicted epitopes of three proteins (blue, yellow, and purple) linked with linkers AAY (green). (b) Physical properties of the trivalent. (c) 3D model of the trivalent modeled by the I-TASSER online server.

The trivalent construct was formulated by integrating three epitopes VSEHRIQDKDGLFY, LLECADDRADLAKY, and EYILSLEELVNGMY of BTNL2, ALB, and GPC3 proteins. A total of 48 amino acid residues in the trivalent molecule including AAY linkers VSEHRIQDKDGLFYAAYLLECADDRADLAKYAAYEYILSLEELVNGMY were constructed. The trivalent was modeled with a confidence score (*C*score) of -2.34, TM score of0.44 ± 0.14, and RMSD of$7.3\pm 4.2\;\overset{{}^{\circ}}{\text{A}}$. The antigenicity score of the trivalent model was 0.69 (Figure 4).

### 3.5. Molecular Docking

**Figure 5:**
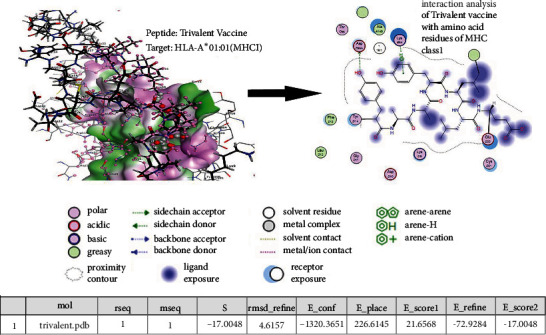
Molecular docking analysis of the trivalent construct with the target HLA-A∗01:01 allele of MHC class 1.

The quality and reliability of the predicted epitopes and trivalent construct were estimated by their 3D models. 3D models were used to reveal their binding affinities with MHC class I molecules. MOE software was used to predict the binding affinities between the trivalent construct and the MHC class I molecules (allele: HLA-A∗01:01). A significant binding affinity of -17.004 kcal/mol was observed between the trivalent construct and the target protein (Table 3) involving the residues SER54, HIS151, ASN53, ASP27, GLN155, GLU154, LEU28, VAL158, TYR34, ALA33, THR95, LYS64, ALA49, ASP95, PHE13, TYR14, LYS9, GLU20, CYS21, ASP10, GLY11, and LEU12 of MHC class I target molecules (Figure 5).

### 3.6. Verification and Assessments of Potential Epitopes

The epitopic sequence and physicochemical properties of potential peptides verified by different tools and software indicated the uniformity and consistency in the sequence and properties (Tables 4 and 5).

### 3.7. Immunoassays and Serological Assays

The activity of potential epitopes was investigated by *in vivo* animal studies with and without an adjuvant. Immune response to synthetic peptides was analyzed 3 weeks postimmunization of rats. The rats were monitored for 3 weeks daily to observe the side effects of the administrated doses. Negative phenotypic signs indicated that there were no side effects of the trivalent peptides (Table 6).

### 3.8. Hematological Assays

**Figure 6:**
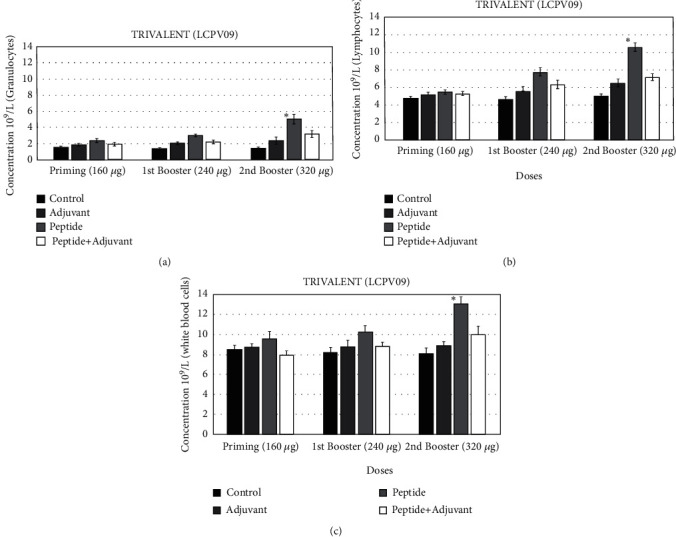
Hematological results were recorded after three weeks of immunization of rats with LCPV09. Blood components were counted using Beckman Coulter, USA. The significance has been shown by ∗*p* < 0.01 ± SEM. (a) Granulocytes. (b) Lymphocytes. (c) White blood cells.

We examined the blood component analysis after immunization. The efficacy of the trivalent (LCPV09) was tested with or without the adjuvant using the rat model. Hematological assays show the primary response to peptides. To compare with the sampled animals, control rats were used and kept under the same conditions. Substantial increases in the count of blood components were recorded. The second booster dose of 320 *μ*g indicated significant results with the adjuvant (*p* < 1.0 × 10^−4^). Significance was found in samples immunized with peptide alone compared to peptides in combination with aluminum hydroxide as an adjuvant. Higher numbers of lymphocytes, white blood cells, and granulocytes (10, 13, and5 × 10^9^/l, respectively) were observed compared to the control (Figure 6).

### 3.9. Liver Function Tests

**Figure 7:**
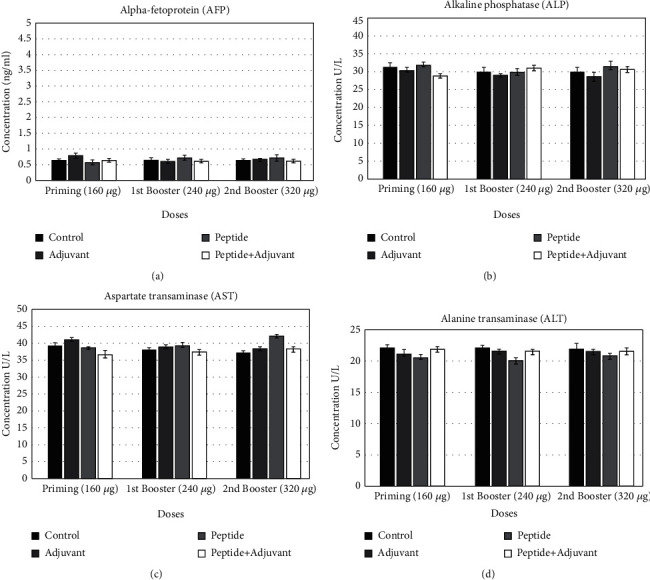
Liver function tests (LFTs) were performed after immunization of rats with each dose. (a) Alpha-fetoprotein (AFP). (b) Serum alkaline phosphatase (ALP). (c) Aspartate transaminase (AST). (d) Alanine transaminase (ALT).

At given doses of trivalent peptides, liver toxicity and function were not disturbed and the level of enzymes was comparable to the controls indicating the safety of these peptides (Figure 7).

### 3.10. IgG ELISA

**Figure 8:**
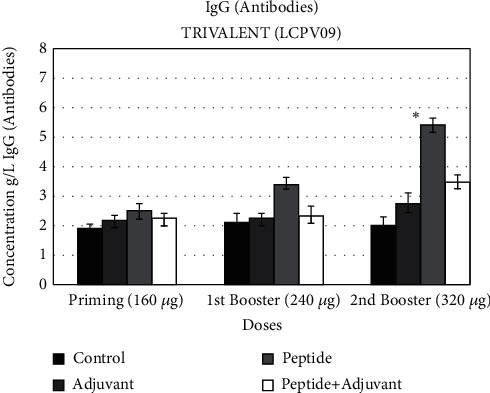
IgG antibodies (g/l). ELISA after each dose by taking four groups of rats. Each group has 6 replicates. Significant results were observed at the second booster dose of LCPV09. The significance has been shown by ∗*p* < 0.01 ± SEM.

The level of IgG was tested after 3 weeks in the blood serum of the immunized rats. We observed the significant IgG production at the second booster dose of 320 *μ*g. The concentration of IgG increased to 6 g/l after taking the peptide without the adjuvant compared to the control (2 g/l) (*p* < 1.0 × 10^−4^), while initial doses of the trivalent showed lower antibody production (Figure 8).

### 3.11. Granzyme B ELISA

**Figure 9:**
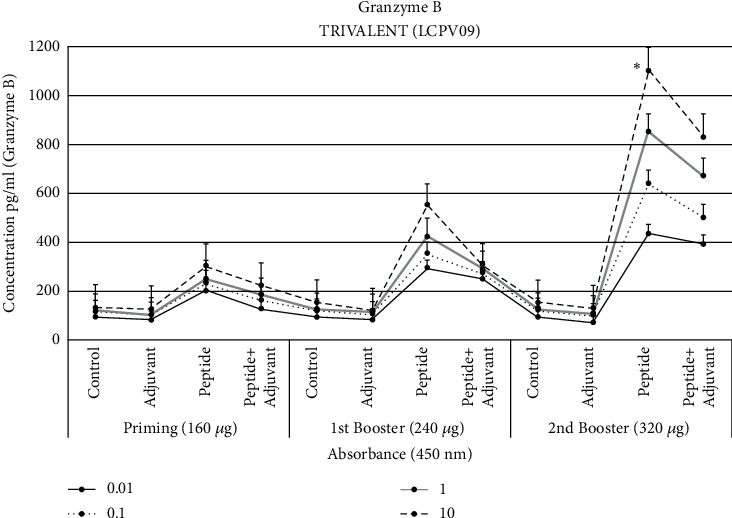
Granzyme B assay was performed, and absorbance was taken at 450 nm after each dose of LCPV09 by taking four groups of rats. Each group has 6 replicates. Significant results were observed at the second booster dose. The significance has been shown by ∗*p* < 0.01 ± SEM.

Due to the immune response, cytotoxic T lymphocytes become activated and secreted granzymes and perforins to destroy cancerous cells through apoptosis [48]. The concentration of granzyme B (GrB) in blood serum was analyzed by measuring the absorbance at 450 nm. Granzyme concentration significantly increased to 1050 pg/ml at the second booster dose of peptides compared to the control (95 pg/ml). Statistical studies revealed that in the serum of trivalent-treated rats, a significant level of GrB was found at (*p* < 1.0 × 10^−4^) ± SEM (Figure 9).

## 4. Discussion

Liver cancer is becoming a great risk to human health due to its increased rate of recurrence, mortality, and metastatic nature [55]. To overcome conventional treatment issues, peptide vaccines have been in consideration for many years despite a few clinical trials. Considering all these aspects, vaccines related to reverse vaccinology have gained attention in cancer treatment [56]. We reported monovalent peptides in our previous studies [17] indicating substantial immune response; however, the present analysis showed the efficacy of trivalent peptides designed from liver-specific antigenic proteins. We observed that these trivalent peptides are antigenic and increased the titer rate of IgG antibodies, granzyme B, interferons, and other chemical mediators. These results indicate that different epitopes of various liver-specific proteins are effective for liver cancer disease. Reverse vaccinology is a cost- and time-effective approach [57], and it has been reported that the GVAX vaccine is the only FDA-approved therapeutic vaccine for prostate cancer designed by this approach. The therapeutic vaccines for pancreatic, lung, breast, and renal cancers are clinical trials [58]. Cancer peptide vaccines composed of small amino acids linked by peptide bonds are an initiative towards anticancer drug development [59]. A vaccine is a noteworthy defining factor in clinical trials. Previous experimental trials were focused on the treatment of predictable cancers. However, in massive tumor disease, the potential immune response is depressed due to progressive tumors, which ultimately leads to immunosuppression. Emerging technology has been focused on low or no symptomatic diseases as a result of immunization response. A distinct way of vaccine immunization is peptide-based vaccines. T cells provoke immunity against cancer cells, which may be direct or indirect by inducing adaptive or innate immunity. Antibody response in immunity can be ducked by antigenic drift over time, while cell-mediated immunity is long-term immunity with memory [60].

In our study, we used tissue-specific antigenic proteins that are highly expressive in liver cancer compared to the BORIS cancer-testis antigen and hydatid cyst wall antigens that are not expressive in liver cells and may not have a role in the treatment and designing of liver cancer vaccines [61, 62]. Although these parasite antigens showed substantial immunogenicity and high epitope homology with cancer antigens, however, these proteins are not expressed in the liver [62]. We used protein data related to specific liver cancer to find potential vaccine candidates that can improve existing vaccine specificity. There may be a risk of occurrence of any side effect in the body by using hydatid cyst wall antigens and nonspecificity of the recognition pattern of liver cancer cells. The antitumor effects of protein cancer vaccines were assessed in metastatic nonimmunogenic 4T1 mammary carcinoma in BALB/c mice compared to this study that was carried out in Sprague Dawley rat models presenting increased serum levels of IgG and interferon-*γ*. Our method of prediction is generalized and can be applied to infectious diseases and genetic disorders [63].

Many cancer vaccines have recently been designed to prevent the spread of cancer. To produce multiepitope DNA and peptide cancer vaccines, different bioinformatics techniques were used to determine the most immunodominant epitopes of acrosin-binding protein (ACRBP) and synaptonemal complex protein 1 (SYCP1) antigens. The peptide vaccination resulted in a considerable rise in serum IgG antibodies and interferon levels. In the murine melanoma model, the results show that the proposed multiepitope peptide vaccination has high effectiveness for immune system activation and antitumor preventive effects [64]. In the following study, the potential of antigenic epitopes to stimulate the immune system against liver cancer was studied. Liver cancer proteins were screened, and their antigenicities were predicted. The extracellular matrix and membrane-bounded antigenic proteins of higher molecular weight > 23 kDa were selected for the activation of immune components. Through the different steps of bioinformatics approaches, the three best antigenic proteins ALB, GPC3, and BTNL2 were screened. These proteins have high expression in the liver, and GPC3 has already gone under clinical trials [50]. Epitopes of length 14 amino acid residues were predicted indicating a maximum binding affinity for MHC class I molecules. The potential trivalent vaccine candidate is designed from these three proteins and synthesized for the generation of an effective immune response. To prevent any alteration in epitope arrangement and functionality, AAY linkers were added to a trivalent containing 48 amino acid residues. In *in vivo* analysis, the predicted trivalent vaccine stimulates cytotoxic T lymphocytes because of their affinity to receptors of MHC class I. Analysis of the interaction and function associations between predicted epitopes and other proteins also describes their significance in liver cancer immunotherapy. Rat models were used to study the immune responses. Hematological immune system components such as granulocytes, lymphocytes, and white blood cells showed substantial results as an immunotherapeutic response at the second booster dose of the trivalent adjuvant. The raised count of lymphocytes, white blood cells, and granulocytes was compared with controls indicating a significant immune response. Similarly, the production of IgG antibodies is secreted as a humoral immune response. The increased concentration of IgG compared to the controls presented substantial outcomes (*p* < 1.0 × 10^−4^).

The *in vitro* cytotoxicity test revealed the granzyme presence in the supernatants. Granzyme concentration significantly increased (1050 pg/ml) at the second booster dose compared to the control. Cytotoxic cells become activated, and granzymes are released when target cells activate them as a usual immune response [63]. NK cells and cytotoxic cells also release granules on stimulation through foreign molecules, and these granules contain granzymes within them [65].

Antigens associated with these tumors open up new opportunities for cancer immunotherapy. This approach helps us to classify possible natural candidates for unique vaccines for customized cancer [63].

## 5. Conclusion

Immunoinformatics is helpful in enhancing the potential of computational practices through which vaccines designed to fight against cancer become more important. Different cancerous antigenic peptides were identified by means of computational methods that offer an advantage for effective vaccine development against liver cancer. The trivalent construct may be a potential vaccine candidate to bind MHC class I molecules. The animal models' different experiments were applied for the confirmation of the effectiveness of these epitopes that are involved in the designing of a vaccine against liver cancer. It is concluded that the design of T cell epitope-based vaccines for liver cancer through the immunoinformatics approach is efficient, practical, cost-effective, and time-effective. The revolutionary changes in the field of reverse vaccinology can be used in disease treatment of both humans and animals as the basic protocol for the design of vaccines using bioinformatics tools.

## Figures and Tables

**Table 1:** List of databases/servers used in the research work for the screening of liver cancer proteins and prediction of epitopes.

Sr. #	Tools/servers/databases	Purpose	Web links	References
1	Human Protein Atlas	Human cancer database	http://www.proteinatlas.org	[26]
2	Bioinformatics and Research Computing	For protein comparisons	http://jura.wi.mit.edu/bioc/tools/compare.php	[66]
3	UniProtKB (UniProt Knowledgebase)	Protein database	https://www.uniprot.org/	[67]
4	CELLO	Subcellular localization prediction	http://cello.life.nctu.edu.tw/	[28]
5	VaxiJen 2.0	Antigenicity prediction	http://www.ddg http://pharmfac.net/vaxijen/VaxiJen/VaxiJen.html	[32]
6	ANTIGENpro	—	http://scratch.proteomics.ics.uci.edu/explanation.html	[68]
7	Immunomedicine Group	—	http://imed.med.ucm.es/Tools/antigenic.html	—
8	CamSol	Solubility prediction	http://www-vendruscolo.ch.cam.ac.uk/camsolmethod.html	[69]
9	SOLpro	—	http://scratch.proteomics.ics.uci.edu/explanation.html#SOLpro	[70]
10	PROSO	—	http://mbiljj45.bio.med.uni-muenchen.de:8888/proso/proso.seam	[69]
11	Immune Epitope Database (IEDB)	Epitope prediction	http://tools.iedb.org/main/	[71]
12	HLAPred		http://crdd.osdd.net/raghava/hlapred/ref.html	[72]
13	ProPred		http://crdd.osdd.net/raghava/propred1/	[73]
14	ToxinPred	Physicochemical properties	https://webs.iiitd.edu.in/raghava/toxinpred/design.php	[36]
15	ProtParam	—	https://web.expasy.org/protparam/	[74]
16	PlifePred	—	https://webs.iiitd.edu.in/raghava/plifepred/index.php	[75]
17	AllergenFP	Allergenecity prediction	http://www.ddg-pharmfac.net/AllergenFP/	
18	AlgPred	—	http://crdd.osdd.net/raghava/algpred/submission.html	[76]
19	PDB (Protein Data Bank)	Protein database	http://www.rcsb.org/pdb/home/home	[77]
20	PEP-FOLD server	Epitope modeling	http://bioserv.rpbs.univ-paris-diderot.fr/PEP-FOLD/	[78]
21	I-TASSER	Protein modeling	https://zhanglab.ccmb.med.umich.edu/I-TASSER/	[79]
22	Molecular Operating Environment	HLA epitope binding prediction	—	—

**Table 2:** Physicochemical properties of the individual epitopes and trivalent construct.

UniProt ID	Peptide sequence	SVM score	Toxicity prediction	Hydropathicity	Charge	Half-life	Instability index
ALBU_HUMAN	LLECADDRADLAKY	-0.36	Nontoxin	-0.31	-2	5.5 hours	23.16
BTNL2_HUMAN	VSEHRIQDKDGLFY	-1.17	Nontoxin	-0.91	-0.5	10 hours	48
GPC3_HUMAN	EYILSLEELVNGMY	-0.57	Nontoxin	0.3	-3	1 hour	52
TRIVALENT	VSEHRIQDKDGLFYAAYLLECADDRADLAKYAAYEYILSLEELVNGMY	-0.84	Nontoxin	-0.221	-6.5	100 hours	34.49

**Table 3:** Screening parameters of the predicted T cell epitopes and trivalent construct.

Sr. no.	EPITOPE_HLA (target)	Sequence	Antigenicity	M/W (kDa)	Solubility	Immunogenicity	Binding energy (kcal/mol)
1	ALB_MHC class I, allele 1	LLECADDRADLAKY	1.0188	1.59579	0.984845	-0.00437	-11.3124
2	BTNL2_MHC class I, allele 1	VSEHRIQDKDGLFY	0.5641	1.69276	0.904257	0.23389	-10.6156
3	GPC3_MHC class I, allele 1	EYILSLEELVNGMY	0.7682	1.7159	0.833942	-0.02795	-8.4656
7	TRIVALENT_MHC class I, allele 1	VSEHRIQDKDGLFYAAYLLECADDRADLAKYAAYEYILSLEELVNGMY	0.6920	5.55021	0.669752	-0.63436	-17.0048

**Table 4:** Verification of the peptide/epitopic sequence and antigenicity using different databases.

UniProt ID	Peptide sequence	Software		
		IEDB [70]	HLAPred [71]	ProPred [72]
ALBU_HUMAN	LLECADDRADLAKY	Antigenic peptide	Antigenic peptide	Antigenic peptide
BTNL2_HUMAN	VSEHRIQDKDGLFY	Antigenic peptide	Antigenic peptide	Antigenic peptide
GPC3_HUMAN	EYILSLEELVNGMY	Antigenic peptide	Antigenic peptide	Antigenic peptide

**Table 5:** Verification of physicochemical properties of predicted peptides using different software.

Properties	Software		
Toxicity	ToxinPred [54]	ProtParam [73]	—
Hydropathicity	ToxinPred	ProtParam	PlifePred [74]
SVM	ToxinPred	ProtParam	—
Charge	ToxinPred	ProtParam	PlifePred
Molecular weight	ToxinPred	ExPASy	PlifePred
Allergenecity	AlgPred [75]	AllergenFP	—
Antigenecity	VaxiJen [32]	Immunomedicine Group	ANTIGENpro [67]
Subcellular localization	CELLO [79]	UniProtKB [66]	—
Solubility	CamSol [68]	SOLPro [69]	PROSO [68]

**Table 6:** Phenotypic signs/symptoms observed after immunization of rats with synthetic peptides.

Sr. #	Signs/symptoms observed	Presence (yes)/absence (no)
1	Inflammation	No
2	Swelling around eyes	No
3	Bleeding	No
4	Diarrhea	No
5	Vomiting	No
6	Dizziness	No
7	Redness/swelling or pain at the site of injection	No
8	Temperature	No
9	Shivery	No
10	Any casualty	Nil

## Data Availability

Data is available in supplementary information files.

## Supplementary Materials

Supplementary Materials Supplementary Table 1: cancer genomic data. Supplementary Table 2: liver proteomic data.
